# A Binocular Vision-Based Crack Detection and Measurement Method Incorporating Semantic Segmentation

**DOI:** 10.3390/s24010003

**Published:** 2023-12-19

**Authors:** Zhicheng Zhang, Zhijing Shen, Jintong Liu, Jiangpeng Shu, He Zhang

**Affiliations:** 1 College of Civil Engineering and Architecture, Zhejiang University, Hangzhou 310058, China; jszzc@zju.edu.cn (Z.Z.); zjshen@zju.edu.cn (Z.S.); lfyer1213@outlook.com (J.L.); jpeshu@zju.edu.cn (J.S.); 2 Center for Balance Architecture, Zhejiang University, Hangzhou 310058, China

**Keywords:** non-contact measurement, crack width, deep learning, image processing, binocular vision

## Abstract

The morphological characteristics of a crack serve as crucial indicators for rating the condition of the concrete bridge components. Previous studies have predominantly employed deep learning techniques for pixel-level crack detection, while occasionally incorporating monocular devices to quantify the crack dimensions. However, the practical implementation of such methods with the assistance of robots or unmanned aerial vehicles (UAVs) is severely hindered due to their restrictions in frontal image acquisition at known distances. To explore a non-contact inspection approach with enhanced flexibility, efficiency and accuracy, a binocular stereo vision-based method incorporating full convolutional network (FCN) is proposed for detecting and measuring cracks. Firstly, our FCN leverages the benefits of the encoder–decoder architecture to enable precise crack segmentation while simultaneously emphasizing edge details at a rate of approximately four pictures per second in a database that is dominated by complex background cracks. The training results demonstrate a precision of 83.85%, a recall of 85.74% and an F1 score of 84.14%. Secondly, the utilization of binocular stereo vision improves the shooting flexibility and streamlines the image acquisition process. Furthermore, the introduction of a central projection scheme achieves reliable three-dimensional (3D) reconstruction of the crack morphology, effectively avoiding mismatches between the two views and providing more comprehensive dimensional depiction for cracks. An experimental test is also conducted on cracked concrete specimens, where the relative measurement error in crack width ranges from −3.9% to 36.0%, indicating the practical feasibility of our proposed method.

## 1. Introduction

Visible cracks in concrete facilitate the unimpeded infiltration of environmental chemicals, such as water, carbon dioxide and chloride ions, thereby promoting corrosion and carbonation [1,2]. When coupled with external loads [3], these durability considerations may exacerbate the occurrence of cracking and result in material discontinuities as well as a localized reduction in structural stiffness [4,5,6,7]. To prevent the functional deterioration of the bridge structure and to mitigate potential safety hazards, periodic crack inspections are essential in assessing the condition of each component and developing appropriate maintenance strategies.

Conventional inspection methods typically involve the use of handheld tools, such as a crack gauge, to detect cracks through direct contact. However, once the inspecting area becomes inaccessible (e.g., the bottom of a beam), heavy machinery like a bridge inspection vehicle is required to provide an operational platform. This entire process is characterized by a high demand for labor, extensive time consumption and substantial costs, while the detected results are susceptible to the inspector’s subjectivity [8,9,10]. To improve this circumstance, several studies have implemented non-destructive testing (NDT) techniques to assist manual inspection. Huston et al. [11], for instance, were able to successfully detect concrete cracks with a width as narrow as 1 mm using a ground penetrating radar (GPR) equipped with a good impedance matching antenna (GIMA). Chen et al. [12] deployed a three-dimensional laser radar, also referred to as 3D LiDAR, to quantify the length of cracking on bridge components, while Valenca et al. [13] incorporated terrestrial laser scanning (TLS) to characterize large-scale structural cracks. In recent years, there has been a growing interest in the utilization of advanced nanomaterials to achieve the self-monitoring of concrete cracks [14,15]. Roopa et al. [16] conducted a study where they incorporated carbon fiber (CF) and multiwalled carbon nanotubes (MWCNT) as nanofillers in the cementitious matrix, aiming to develop self-sensing sensors. These sensors exhibit piezoelectric properties that correspond to the structural response, enabling them to autonomously detect damage. At the microscale, the nanocomposite sensors demonstrate exceptional sensitivity to small cracks, thereby facilitating real-time monitoring of crack formation and propagation. However, it is important to note that this method is relatively susceptible to environmental factors such as temperature and humidity, which can impact its performance. Additionally, while the self-monitoring methods based on nanomaterials can provide estimates of crack width and location, it cannot provide precise information on crack morphology. In general, the exorbitant cost and limited applicability of these abovementioned methods impede their promotion, rendering it arduous to satisfy the demand for crack detection in huge-volume concrete bridges.

Over the past two decades, non-contact, high-precision and low-cost machine vision-based NDT methods have emerged as the potentially viable alternative to manual visual inspection. In this context, camera-mounted unmanned aerial vehicles (UAVs) or robots can function as image sensing-based inspection platforms [17,18,19,20]. The automatic crack detection in large volumes of acquired image data thus poses a significant challenge. Previously, researchers have utilized traditional image processing techniques (IPTs) for crack extraction, proposing hybrid approaches that integrate thresholding, morphological operators or filter concepts [21,22,23,24,25,26,27], as well as approaches based on mathematical transformations [28,29,30,31,32]. A considerable proportion of crack measurements in these studies were conducted on binary images, which can be broadly categorized into three distinct groups. The first group adopts pixel count as a quantitative metric for representing cracks. Payab et al. [33] expressed the crack area and length values in pixel numbers of crack region and skeleton, respectively, and took the ratio of the two as the average crack width. The second type entails a scale factor to convert the output of the first group into actual physical dimensions. After detecting thermal cracks on fire-affected concrete via wavelet transform, Andrushia et al. [34] adopted the unit pixel size, i.e., pixel resolution, to convert the morphological characteristics from pixel units to physical units. The final category achieves measurement by means of crack reconstruction. Liu et al. [35] employed the structure from motion (SFM) algorithm to conduct 3D reconstruction, enabling not only the acquisition of crack width but also the integration of cracks from multiple perspectives into a unified 3D scene.

The attainment of anticipated outcomes through IPT-based methods suitable for simple cracks (i.e., high contrast and good continuity) is a challenging task due to the presence of diverse noises in actual inspection data, necessitating further enhancement in their robustness [36]. Therefore, modified solutions in combination with machine learning (ML) have been proposed. Specifically, the image features extracted by IPTs pass through the supervised learning-based classifier to determine whether they are indicative of a crack. The study conducted by Prasanna et al. [37] focused on the detection of noise-robust line segment features that accurately fit cracks. They employed support vector machines, Adaboost and random forests as classifiers, utilizing spatially tuned multi-feature appearance vectors. The performance of various feature combinations was evaluated, demonstrating that integrating multiple design features into a single appearance vector yields superior classification results. Peng et al. [38] developed a cascade classifier for determining the positivity and negativity of crack detection windows by extending diverse Haar-like features and employed a monocular vision technique, which belongs to the second category of measurement methods, to calculate the actual crack width. While the incorporation of ML into such methodologies strengthens their adaptability to real-world scenarios, it is inevitable that the results will still be influenced by IPTs.

Deep learning (DL) is an emerging and powerful alternative to the above methods, with the advantage of not depending on expert-dominated heuristic thresholds or hand-designed feature descriptors, thereby greatly enhancing the accuracy and robustness of feature extraction [39]. During recent years, a multitude of researchers have extensively investigated the potential of DL-based models, particularly convolutional neural networks (CNNs), for concrete crack detection. The aforementioned studies demonstrated successful applications of CNNs in image classification [40] and object identification tasks, specifically pertaining to crack detection at both the image level/patch level [41,42,43,44] and object level [45,46,47]. However, neither the grid-like detected results nor the bounding boxes with class labels provide a precise description of the crack topology. In contrast, semantic segmentation categorizes each pixel into a possible class (e.g., crack or background), offering the highest level of detail in features. To detect cracks at the pixel level, Li et al. [48] trained a CNN-based local pattern predictor for coarse analysis on crack pixels. Kim et al. [49] adopted Mask R-CNN for instance segmentation of concrete cracks but not complete semantic segmentation, hence having limited precision. Zhang et al. [50] developed CrackNet-R, an effective semantic segmentation network for detecting cracks in asphalt pavement but also prone to technical isolation in practice.

With the widespread adoption of the encoder–decoder architecture in semantic segmentation, various CNNs have been proposed for pixel-level crack detection based on different variations of this structure, including fully convolutional network (FCN) [51,52], U-Net [53,54,55,56], SegNet [57,58,59], DeepLab series [60,61] and ResNets [62,63]. These architectures consist of two components, namely the encoder module responsible for extracting multi-scale features and the decoder module dedicated to restoring the feature information. On the one hand, the decoders upscale the final output of the encoder network to match the original input size, thereby facilitating the orientation of crack pixels. On the other hand, the encoders supply the local information during the decoding process to minimize loss of details from the input. Although the mentioned classical neural networks demonstrate proficiency in executing fundamental segmentation operations, they remain confronted with difficulties in achieving precise object edge segmentation and addressing class imbalance. Consequently, researchers have started integrating various cutting-edge methods to optimize the performance of segmentation models. In light of the requirement for both semantic understanding and fine-grained detail in segmentation tasks, a suite of attention-based methodologies [64,65] have been developed. These methods are designed to assimilate multi-scale and global contextual information, thereby enhancing the accuracy of defect identification. Chen et al. [66] have demonstrated impressive recognition accuracy in identifying different types of cracks by incorporating the Convolutional Block Attention Module (CBAM) into MobileNetV3 as the backbone network. Du et al. [67] have proposed an Attention Feature Pyramid Network that enhances the precise segmentation of road cracks within the YOLOv4 model. Similarly, Yang et al. [68] introduced a multi-scale, tri-attention network, termed MST-NET. Other advanced computational modules, such as separable convolution [69] and deformable convolution [70], have been introduced to further enhance model performance. Recognizing that the training of semantic segmentation models heavily relies on accurately annotated data, numerous researchers have also begun exploring approaches to enhance the generalization and adaptability of segmentation methods from the perspective of dataset optimization and learning strategies. For instance, Que et al. [71] have proposed a crack dataset expansion method based on generative adversarial networks (GANs), resulting in higher recall rates and F1 scores for the same model. Nguyen et al. [72] have introduced the Focal Tversky loss function to tackle class imbalance issues in crack segmentation, shedding light on the role of loss functions during model training. Furthermore, Weng et al. [73] have devised an unsupervised adaptive framework for crack detection, effectively mitigating domain shift problems among various civil infrastructure crack images.

On this basis, the first category of crack measurements was completed by Yang et al. [51], Ji et al. [60] and Kang et al. [74]. Regrettably, these results are inadequately cited for crack evaluation purposes. To make sense of the measure values, Li et al. [36] and Chen et al. [65] employed a monocular vision technique to accurately quantify the crack indicators such as area, max width and length. However, these methods rely on calibrated pixel resolution and the similar triangle relationship for unit conversion, which necessitates frontal photography of the target crack at known distances with a monocular device. As a result, restricted shooting postures increase the difficulty of remotely manipulating inspection platforms, leading to complications in image acquisition and unstable measurements.

The third category of binocular stereo vision-based measurement emerges as a promising solution to tackle the aforementioned challenges. In contrast to monocular vision, which calculates physical dimensions mapped on pixels, binocular stereo vision reconstructs the 3D coordinates of a crack in a datum coordinate system based on internal imaging geometries and the external relative posture of two cameras, as well as matching relations between two captured images. This enables a more comprehensive and reliable quantification of morphological characteristics. Furthermore, binocular vision is not constrained by a fixed photogrammetric geometry and offers greater flexibility in capturing cracks within its depth of field. Previously, Guan et al. [56] designed a vehicle-mounted binocular photography system to generate 3D pavement models and precisely estimated the volume of pavement potholes by integrating pixel-level predictions of a U-Net but failed to further quantify the segmented cracks. Yuan et al. [75] and Kim et al. [76] upgraded the automation of non-contact inspection through a robot and a UAV equipped with binocular devices, respectively, despite their crack predictions not being derived from semantic segmentation networks. Recently, Chen et al. [77] optimized DeeplabV3+ to deliver a detailed crack morphology for measurement based on binocular stereo vision, resulting in satisfactory outcomes.

In this paper, a novel non-contact crack detection and measurement method in combination with an encoder–decoder FCN and binocular stereo vision is proposed for efficient and accurate evaluation of concrete cracks in bridge structures. The proposed method not only enhances the flexibility of crack data acquisition but also enables rapid and precise extraction of crack morphology, which facilitates 3D reconstruction in the form of spatial discrete points, thereby obtaining a more comprehensive set of dimensional information regarding cracks. The limitations on shooting attitude imposed by the monocular measurement method are thus effectively addressed, along with the issues related to accuracy and robustness in traditional crack detection methods. Moreover, in contrast to conventional binocular vision-based 3D reconstruction methods that rely heavily on feature matching prior to point cloud computation, the proposed method employs projective reconstruction, which significantly alleviates computational expenses and eliminates potential mismatches between the two views.

## 2. Methodology

### 2.1. Overview

**Figure 1:**
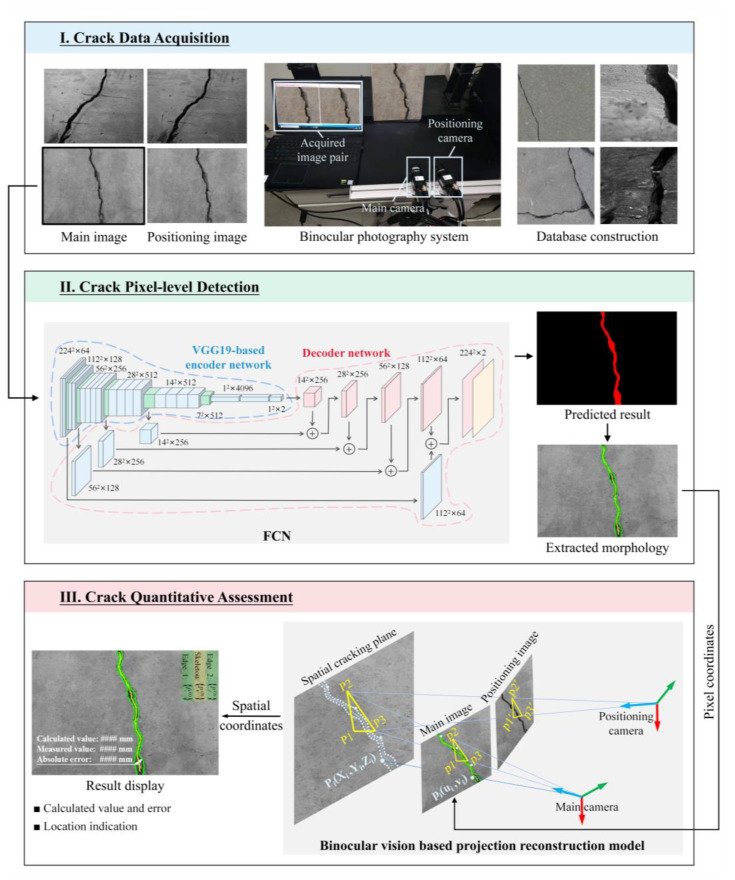
The overall workflow of the method. (The # represents the specific numerical results for different cracks.).

The proposed method consists of three parts, as depicted in Figure 1, which illustrates the overall workflow schematically. (I) Crack data acquisition: a tailored binocular system is constructed for capturing visible cracks from multiple angles at flexible distances, rendering it ideal for UAV-aided crack inspection. The captured image pairs subsequently serve as primary data to detect cracks. (II) Crack pixel-level detection: to achieve precise segmentation of cracks in the main images from primary data, a semantic segmentation network (i.e., the encoder–decoder FCN) is constructed with a VGG19-based encoder network and a decoder network featuring the deconvolution layer as its core. The resulting binary image is further exploited to extract pixels that characterize the morphology of the crack. (III) Crack quantitative assessment: at this stage, a binocular vision-based projection reconstruction model is employed for spatial localization of the cracked concrete surface and subsequent 3D crack reconstruction by projecting pixels extracted in the previous stage onto it. Finally, the morphological characteristics of cracks are quantitatively calculated based on the discrete reconstructed points. A detailed description of each part is presented below.

### 2.2. Crack Data Acquisition

**Figure 2:**
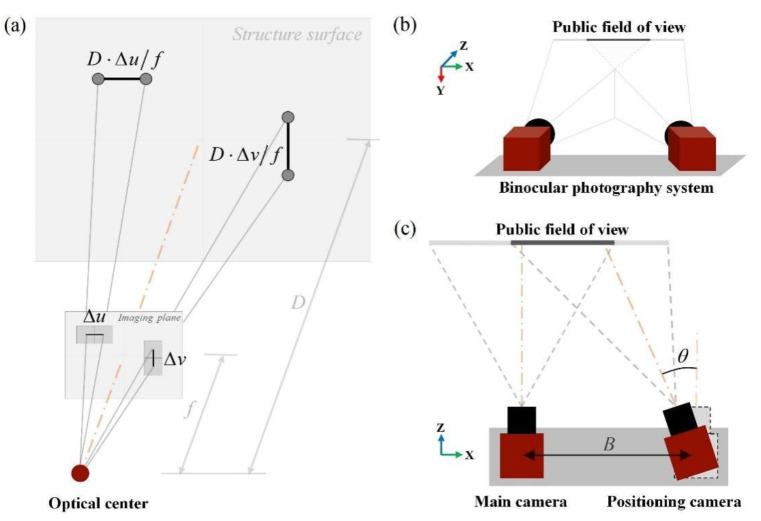
Considerations of the binocular system: (**a**) a pinhole model for resolution and distance trade-off; (**b**) public field of view of two specifically mounted cameras; and (**c**) overhead perspective of (**b**).

To facilitate the UAV assistance, a pair of identical industrial charge-coupled device (CCD) cameras from Microvision, a supplier specialized in visual products, are rigidly assembled for a lightweight and compact binocular photography system. The specifications for each component are comprehensively presented in Table 1, where the outgoing focal length *f* is 16 mm, with a pixel size ∆*u*·∆*v* of 3.75 × 3.75 μm^2^. According to the pinhole model depicted in Figure 2a, the resolution of a single camera at an operating distance *D* of 200 ± 50 mm is approximately 0.047 ± 0.012 mm/pixel, which is adequate for capturing crack details. Moreover, to take into account the public field of view (Figure 2b), the relative pose of two cameras is adjusted with a narrow baseline (denoted as *B* and set to 5 cm) and the intersecting optical axes (realized by a left deviation of the right camera at angle *θ* of roughly 20°), as shown in Figure 2c. For the subsequent description, the left camera is designated as the main camera along the shooting direction, while the right camera is designated as the positioning camera. These two cameras capture images of target cracks synchronously to form stereo image pairs, which are then transmitted in real time to the inspector’s laptop.

### 2.3. Crack Pixel-Level Detection

The accurate and efficient characterization of crack morphology is a prerequisite for real-time image measurement of concrete cracks. To accomplish this, a specialized encoder–decoder FCN is developed for detecting cracks at the pixel level. Subsequently, an integrated computer vision (CV) program is written to enable rapid extraction of the edges and skeletons that characterize the crack morphology from the FCN predictions.

#### 2.3.1. FCN for Crack Segmentation

**Figure 3:**
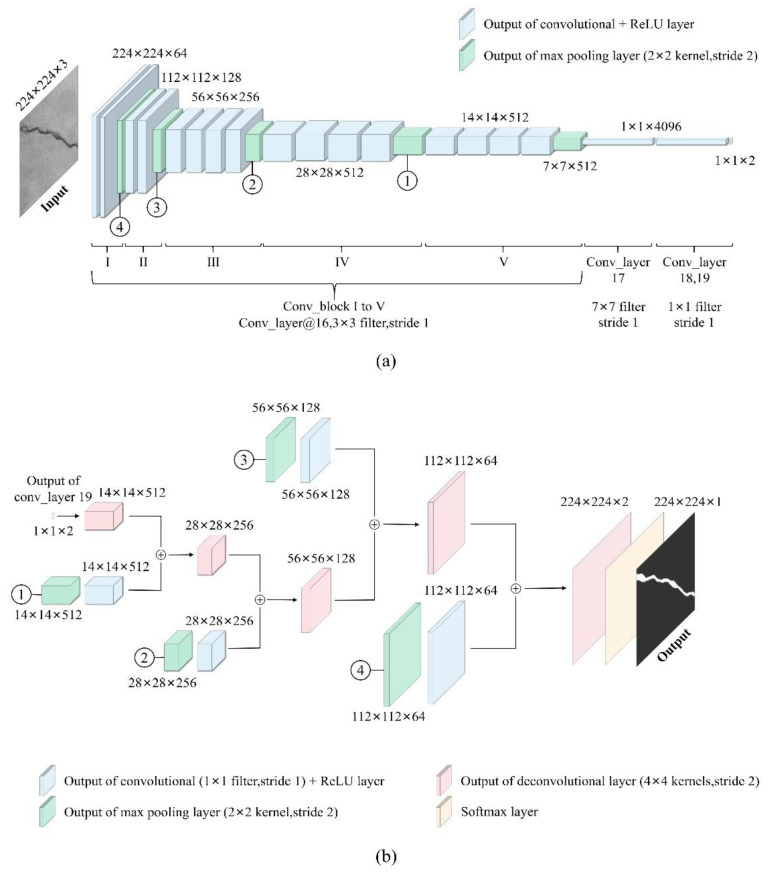
(**a**) Encoder network and (**b**) decoder network of FCN.

The state-of-the-art CNNs, such as VGG16 [52], ResNet [62] and DenseNet [36], which serve as the encoder of FCNs for robust feature extraction in crack images, directly inspire the construction of the FCN framework in this study. Among these classical CNN models, the VGG series, including VGG16 and depth-increased VGG19, are extensively applied for large-scale image detection tasks due to their good transferability. Considering that employing transfer learning [78,79] based on pre-trained parameters of VGG can not only significantly reduce the overall training time of the FCN model but also effectively enhance its performance in scenarios with limited training data, the VGG19-based encoder network is adopted to extract essential features for semantic segmentation. As shown in Figure 3a, the encoder network is topologically identical to the first 16 layers of VGG19, consisting of five convolutional blocks (also referred to as encoders in this paper) that include all convolutional layers, nonlinear activation layers utilizing the ReLU function and pooling layers. Since the encoder module does not involve neuron classification, the final softmax layer of VGG19 is excluded, while the fully connected layers are replaced by convolutional layers with two dropout layers added in between to prevent overfitting.

Inheriting the strengths of VGG19, each encoder conducts convolution operations through the stacking of 3 × 3 filters (i.e., convolution kernels) with a fixed stride length of 1 pixel, which ensures the equivalent receptive field as larger-size filters, while extracting higher-level features with fewer parameters of the convolution kernel. Moreover, ReLU activation is applied following each convolution to introduce nonlinearity, thereby enhancing the nonlinear fitting capability of the encoder network. To eliminate redundant information and to accelerate computational speed, the max pooling operation is subsequently performed on a 2 × 2 pixel window with a stride of 2, which results in downsampling of the output by a factor of 2. It is noteworthy that the outputs of the first four max pooling layers, numbered ④, ③, ② and ①, will also be recycled by the decoder network. Due to the three newly substituted convolution layers, namely Conv_layer 17,18 and 19, the final output is transformed from the initial class probabilities into a low-resolution feature map that characterizes the crack, which is subsequently fed into the decoder module.

The decoder network employs deconvolutional upsampling to generate a dense output and rescales the data to the original input size. To minimize the loss of details during the decoding process, the skip connection structure proposed by Bang et al. [62] is adopted to facilitate the flow of feature maps from the upstream encoders to their corresponding downstream counterparts, which enables effective integration of multi-scale and multi-level local information. Specifically, each decoder selectively fuses the local feature map with the upstream feature map at the expense of increased memory consumption. 

Referring to the decoder network depicted in Figure 3b, the max pooling outputs labeled as ①, ②, ③ and ④ are initially individually convolved with a 1 × 1 kernel for densification purposes. The subsequent outputs are considered to hold local information originating from the upstream network (i.e., the encoder network) and are then arithmetically added (represented by “⊕” in Figure 3b) to the upsampling results of identical resolution obtained through deconvolution with a 4 × 4 kernel with a two-pixel stride. The entire decoder network integrates the outputs from the final layer and the first four max pooling layers of the encoder network, wherein each fused feature map undergoes a doubling in resolution through upsampling with a stride of 2. After five upsamplings, the output of conv_layer 19 is expanded to match the dimensions of the original input and then proceeds through the softmax layer, where the softmax function value determines the probability of a single pixel belonging to either the “crack” or “background” categories. Ultimately, a binary image is exported as the final prediction, with “crack” pixels assigned a value of 1, while the “background” pixels assigned a value of 0.

#### 2.3.2. Extraction of Crack Edges and Skeletons

**Figure 4:**
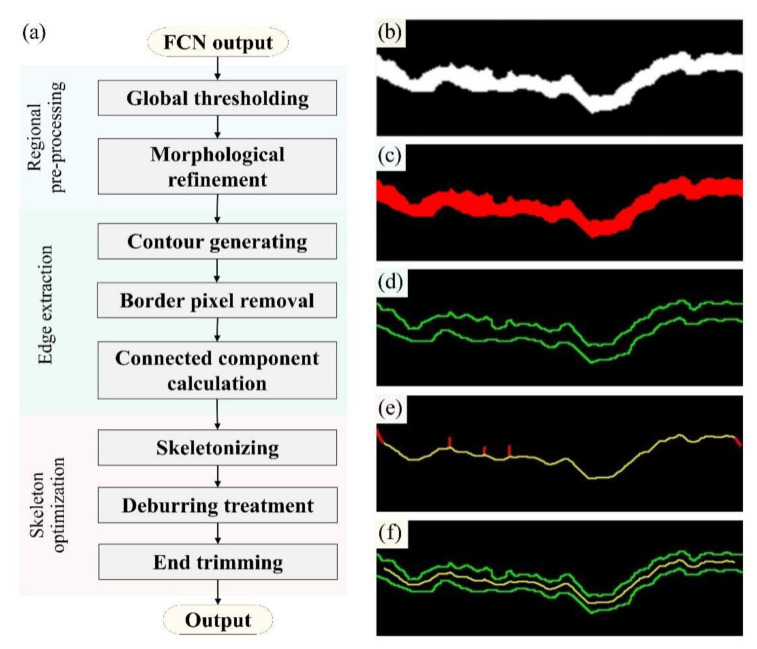
Procedures for crack edge and skeleton extraction: (**a**) flow chart; (**b**) FCN prediction; (**c**) refined crack region; (**d**) crack edges; (**e**) original crack skeleton (The red lines represent the pruned excess crack branches and the yellow lines represent the crack skeletons.); and (**f**) outputs of crack morphology.

The CV procedure for crack edge and skeleton extraction consists of three stages: region pre-processing, edge extraction and skeleton optimization (Figure 4a). Firstly, the FCN prediction shown in Figure 4b is subjected to global segmentation using a fixed threshold of 180 as an empirical value. This procedure successfully eliminates isolated data points outside the cracks. In addition, a morphological optimization technique is employed, which entails the sequential application of dilation and erosion. After this step, marginal burrs and internal holes caused by misjudgment of the proposed FCN can be effectively eliminated. Figure 4c presents the optimized crack region. Secondly, the contour extraction technique in OpenCV is subsequently applied to acquire the single-pixel-wide crack edges. Given that the image boundary truncates the crack and forms a closed contour along with its edges, it becomes imperative to exclude the boundary pixels within this contour. The specific solution is to identify the difference set between the crack region and the pixel border of the image. Next, the connected component is calculated, and the remaining regional contours are divided into the two crack edges (Figure 4d).

Finally, the skeleton of the crack region is extracted and optimized using the fast parallel thinning algorithm proposed by Zhang et al. [80]. During this process, the superfluous branches of the original crack skeleton are pruned through deburring treatment. This involves identifying branch nodes and calculating the number of path pixels, which removes branches that fall below a preset threshold and thus retains only the longest path, i.e., the backbone portion of the skeleton. To further mitigate the issue of tail ends of the crack skeleton converging towards the cusp in the crack region, resulting in incongruity with the actual crack topology, as indicated by the red end in Figure 4e, an end trimming treatment is implemented, in which any skeleton part that falls within 20 pixels (based on experience) from the image boundary will be cropped. The final outputs, as presented in Figure 4f, are stored as pixel coordinates.

### 2.4. Crack Quantitative Assessment

The proposed projection reconstruction model consists of a binocular vision model for locating the spatial crack plane and a central projection model for reconstructing the crack morphology. Based on the discrete reconstruction points, the dimensions of concrete cracking in bridge structures can be quantitatively assessed.

#### 2.4.1. Binocular Vision for Crack Location

**Figure 5:**
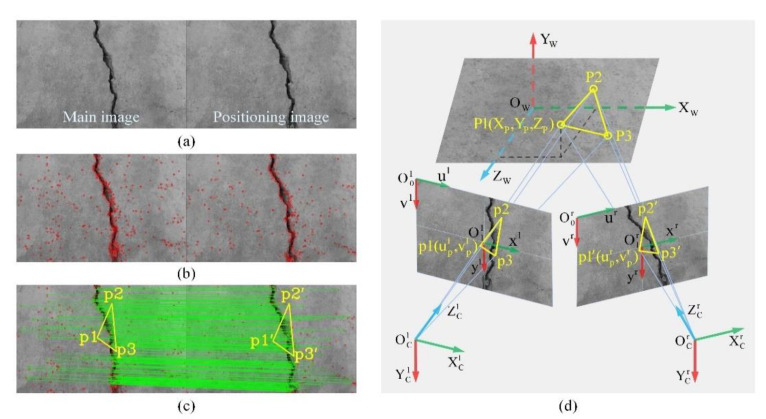
Crack plane location: (**a**) stereo image pair; (**b**) feature point extraction; (**c**) feature point matching with randomly selected three-point pair; and (**d**) binocular vision model to calculate the spatial location points.

Our crack location approach is illustrated in Figure 5. First, the points of interest in a stereo image pair (Figure 5a) are extracted and matched using the correspondence search techniques, as indicated by the red dots in Figure 5b. Each pair of matching points is considered the projection of a specific point on the cracked structure onto both imaging planes, which is connected by a green line in Figure 5c. The next step involves randomly selecting three non-colinear feature points (*p*_1_, *p*_2_ and *p*_3_) in one image, along with their corresponding matching points (*p*_1_’, *p*_2_’ and *p*_3_’, respectively) in another image, to form a three-point pair for the purpose of planar location. Herein, to avoid the selected points falling into the crack region, the contour is dilated by five pixels as the boundary for pre-filtering the internal feature points. Consequently, only feature points located on the background of the image remain. Finally, the binocular vision model depicted in Figure 5d is utilized to calculate the non-collinear spatial location points (*P*_1_, *P*_2_ and *P*_3_) corresponding to the aforementioned three-point pair for achieving the precise localization of the flat concrete surface.

Previously, the scale-invariant feature transform (SIFT) algorithm proposed by Lowe [81] was successfully applied to extract features from crack images [56,82], showcasing its robustness to rotation and translation, as well as its capability to handle variations in lighting conditions and viewpoints. Our approach employs the SIFT algorithm for scale space filtering of stereo image pairs, facilitating the detection of feature points across multiple scales. For the *k*th stereo image pair $I^{\left(k\right)}=\left\{I_{1}^{\left(k\right)},I_{2}^{\left(k\right)}\right\}$, with $I_{1}^{\left(k\right)}$ and $I_{2}^{\left(k\right)}$ representing the k main image and the positioning image, respectively, the extracted feature point sets are denoted as $F_{1}^{\left(k\right)}=\left\{(p_{1,i}^{\left(k\right)},f_{1,i}^{\left(k\right)})|i=1\ldots P\right\}$ and $F_{2}^{\left(k\right)}=\left\{(p_{2,j}^{\left(k\right)},f_{2,j}^{\left(k\right)})|j=1\ldots Q\right\}$, where $f_{1,i}^{\left(k\right)}$ and $f_{2,j}^{\left(k\right)}$ are the local feature descriptors corresponding to feature point positions $p_{1,i}^{\left(k\right)}$ and $p_{2,j}^{\left(k\right)}$, respectively. On this basis, the first two nearest neighbors of $(p_{1}^{\left(k\right)},f_{1}^{\left(k\right)})\in F_{1}^{\left(k\right)}$ are searched with Euclidean distance in the query set $F_{2}^{\left(k\right)}$ by applying the nearest neighbor algorithm. The optimal matches are then obtained through a threshold of 0.5 to the ratio between the Euclidean distances of the nearest and second-nearest neighbors. The matching result is a set of feature point pairs, i.e., $\left\{(p_{1}^{\left(k\right)},p_{2}^{\left(k\right)})|p_{1}^{\left(k\right)}\in I_{1}^{\left(k\right)},p_{2}^{\left(k\right)}\in I_{2}^{\left(k\right)}\right\}$, from which three pairs of location points are randomly selected.

The binocular photography system is simplified into a binocular vision model, as illustrated in Figure 5d. Here, $O_{C}^{l}-X_{C}^{l}Y_{C}^{l}Z_{C}^{l}$ represents the main camera coordinate system (m-CCS), $O_{1}^{l}-x^{l}y^{l}$ and $O_{0}^{l}-u^{l}v^{l}$ denote the physical and pixel coordinate systems on the main image, respectively; the positioning camera coordinate system (p-CCS), i.e., $O_{C}^{r}-X_{C}^{r}Y_{C}^{r}Z_{C}^{r}$, is situated on the right side with the two corresponding image coordinate systems $O_{1}^{l}-x^{l}y^{l}$ and $O_{0}^{l}-u^{l}v^{l}$; and $p1(u_{p}^{l},v_{p}^{l})$ and $p1^{\prime }(u_{p}^{r},v_{p}^{r})$ represent the projected pixels of a specific point $P1(X_{P},Y_{P},Z_{P})$ on the crack plane in world coordinate system $O_{W}-X_{W}Y_{W}Z_{W}$ (WCS), as captured by the two imaging planes, respectively.

Taking point P1 as an example for calculation, assuming WCS coincides with m-CCS, the projection relationship between $P1(X_{P},Y_{P},Z_{P})$ and $p1(u_{p}^{l},v_{p}^{l})$ is given by the following:

(1) $$Z_{P}\left(\begin{array}{c} u_{p}^{l}\\ v_{p}^{l}\\ 1 \end{array}\right)=A_{1}\left[\begin{array}{cc} I_{3} & O_{3\times 1} \end{array}\right]\left(\begin{array}{c} X_{P}\\ Y_{P}\\ Z_{P}\\ 1 \end{array}\right)=\left(\begin{array}{ccc} \begin{array}{c} f^{l}/k^{l}\\ 0\\ 0 \end{array} & \begin{array}{c} \gamma_{1}\\ f^{l}/l^{l}\\ 0 \end{array} & \begin{array}{c} u_{0}^{l}\\ v_{0}^{l}\\ 1 \end{array} \end{array}\right)\left(\begin{array}{c} X_{P}\\ Y_{P}\\ Z_{P} \end{array}\right)$$

where $A_{1}$ is the intrinsic matrix of the main camera, with $f^{l}$ the focal length, $\left(u_{0}^{l},v_{0}^{l}\right)$ the pixel coordinates of the principal point $O_{1}^{l}$, as well as $k^{l}$ and $l^{l}$ the physical length of the pixel unit along the $u^{l}$-axis and $v^{l}$-axis directions, respectively; $\gamma_{1}$ is the parameter characterizing the skew of the two image axes, which is typically zero; $I_{3}$ denotes the 3 × 3 unit matrix, while $O_{3\times 1}$ represents the 3 × 1 zero vector.

The projection formula from $P1(X_{P},Y_{P},Z_{P})$ to $p1^{\prime }(u_{p}^{r},v_{p}^{r})$ is simultaneously established by utilizing the relative pose of the two cameras, as demonstrated below:

(2) $$Z_{P1}\left(\begin{array}{c} u_{P1}^{r}\\ v_{P1}^{r}\\ 1 \end{array}\right)=A_{2}\left[\begin{array}{cc} R & t \end{array}\right]\left(\begin{array}{c} X_{P1}\\ Y_{P1}\\ Z_{P1}\\ 1 \end{array}\right)={\overset{–}{A}}_{2}\left(\begin{array}{cccc} f^{r}R_{11} & f^{r}R_{12} & f^{r}R_{13} & f^{r}t_{x}\\ f^{r}R_{21} & f^{r}R_{22} & f^{r}R_{23} & f^{r}t_{y}\\ R_{31} & R_{32} & R_{33} & t_{z} \end{array}\right)\left(\begin{array}{c} X_{P1}\\ Y_{P1}\\ Z_{P1}\\ 1 \end{array}\right)$$

where $A_{2}$ represents the positioning camera intrinsic matrix, which is structurally and parametrically equivalent to $A_{1}$; ${\overset{–}{A}}_{2}=A_{2}\times \mathrm{diag}(1/f^{r},1/f^{r},1)$, with diag symbolizing the diagonal matrix; and $R={\left[R_{ij}\right]}_{3\times 3}$ and $t={\left[t_{x},t_{y},t_{z}\right]}^{T}$ are the rotation matrix and translation vector, respectively, of the main camera relative to the positioning camera in the binocular system, serving as its external parameters.

From Equations (1) and (2), the spatial coordinates of the point P1 can be obtained:

(3) $$X_{P}=Z_{P}\frac{x_{p}^{l}}{f^{l}}$$

(4) $$Y_{P}=Z_{P}\frac{y_{p}^{l}}{f^{l}}$$

(5) $$\begin{array}{r} Z_{P}=\frac{f^{l}(f^{r}t_{x}-x_{p}^{r}t_{z})}{x_{p}^{r}(x_{p}^{l}R_{31}+y_{p}^{l}R_{32}+f^{l}R_{33})-f^{r}(x_{p}^{l}R_{11}+y_{p}^{l}R_{12}+f^{l}R_{13})}\\ =\frac{f^{l}(f^{r}t_{y}-y_{p}^{r}t_{z})}{y_{p}^{r}(x_{p}^{l}R_{31}+y_{p}^{l}R_{32}+f^{l}R_{33})-f^{r}(x_{p}^{l}R_{21}+y_{p}^{l}R_{22}+f^{l}R_{23})} \end{array}$$

where $\left(x_{p}^{l},y_{p}^{l}\right)$ and $\left(x_{p}^{r},y_{p}^{r}\right)$ are the physical coordinates of the projected pixels $p1(u_{p}^{l},v_{p}^{l})$ and $p1^{\prime }(u_{p}^{r},v_{p}^{r})$, respectively, which can be expressed as follows:

(6) $$\left[\begin{array}{c} x_{p}^{l}\\ y_{p}^{l}\\ x_{p}^{r}\\ y_{p}^{r}\\ 1 \end{array}\right]=\left[\begin{array}{ccccc} k^{l} & 0 & 0 & 0 & -k^{l}u_{0}^{l}\\ & l^{l} & 0 & 0 & -l^{l}v_{0}^{l}\\ & & k^{r} & 0 & -k^{r}u_{0}^{r}\\ & & & l^{r} & -l^{r}v_{0}^{r}\\ & & & & 1 \end{array}\right]\left[\begin{array}{c} u_{p}^{l}\\ v_{p}^{l}\\ u_{p}^{r}\\ v_{p}^{r}\\ 1 \end{array}\right]$$

According to Equations (5) and (6), the mapping relationship between a pair of homologous pixels to its spatial source point is established. With the internal and external parameters obtained from calibration, the location of the cracking plane can be determined in m-CCS.

#### 2.4.2. Central Projection for Crack Reconstruction

The binocular vision model enables spatial point reconstruction, contingent upon feature matching to establish the correspondence between the two views. To alleviate computational expenses and reconstruction errors resulting from mismatches, a projection reconstruction scheme is proposed.

**Figure 6:**
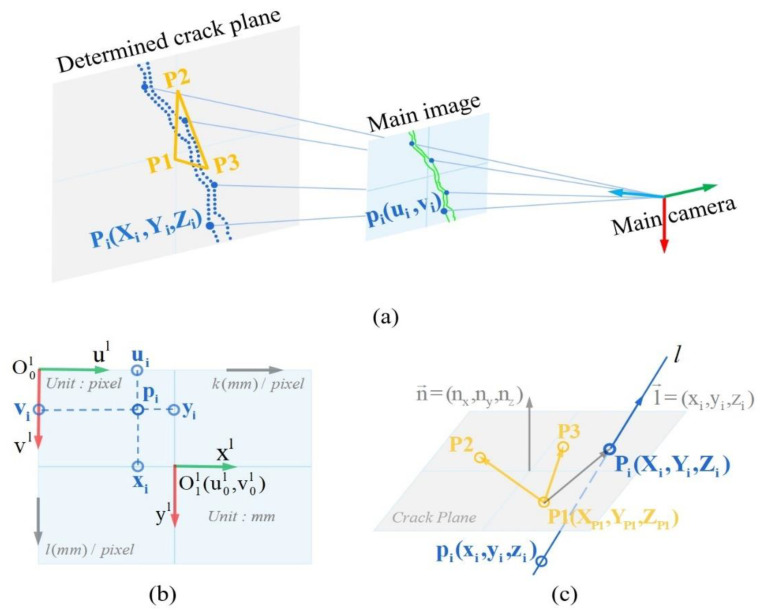
Central projection for crack reconstruction: (**a**) central projection model; (**b**) coordinate transformation on the main image; and (**c**) projection point calculation.

The central projection model is constructed by taking the origin of the main camera model, namely the optical center $O_{C}^{l}$, as the projection center; the determined spatial cracking plane as the easel plane; and the pixels of crack edges and skeleton extracted from the main image as the points to be projected, as shown in Figure 6a. The model achieves 3D reconstruction by mapping pixels from the main imaging plane onto the cracked concrete surface. Prior to this, the reference systems, or the main camera coordinates of target pixels need to be standardized. According to the properties of pinhole camera model, the location of the main imaging plane depicted in Figure 6b under the main camera coordinate system is as follows:

(7) $$Z_{C}^{l}=f^{l}(-\frac{W}{2}-\Delta u\leq X_{C}^{l}\leq \frac{W}{2}-\Delta u,-\frac{H}{2}-\Delta v\leq Y_{C}^{l}\leq \frac{H}{2}-\Delta v)$$

where W and H represent the width and height of the main image, respectively, and (△u,△v) denotes the deviation of the calibrated principal point $O_{1}^{l}(u_{0},v_{0})$ from the image center. Therefore, the $Z_{C}^{l}$-coordinates of all pixels to be projected are numerically equal to the focal length $f^{l}$. Since $O_{1}^{l}-x^{l}y^{l}$ can be regarded as the projection of the $X_{C}^{l}$- and $Y_{C}^{l}$-axes on the main imaging plane, the corresponding camera coordinates of $p_{i}(u_{i},v_{i})$ also represent the physical coordinates of $\left(x_{i},y_{i}\right)$, which can be interconverted by the scale factors $k^{l}$ and $l^{l}$ in the directions of the $u^{l}$- and $v^{l}$-axes, respectively, as well as the origin $O_{1}^{l}(u_{0},v_{0})$, as indicated by Equation (6). The transformation of the target pixel onto the main camera coordinate system is thus given by the following:

(8) $$f:(u_{i},v_{i})\to (x_{i},y_{i},z_{i})=((u_{i}-u_{0})k^{l},(v_{i}-v_{0})l^{l},f^{l})$$

After establishing a unified reference system with Equation (8), the projection points on the easel plane are calculated. As shown in Figure 6c, $\vec{n}=(n_{x},n_{y},n_{z})$ is the normal vector of the spatial cracking plane, determined by vectors $\vec{P1,P2}$ and $\vec{P1,P3}$; the crack pixel $p_{i}(x_{i},y_{i},z_{i})$ serves as a particular point on the projection line $l_{i}$, while ${\vec{l}}_{i}=(x_{i},y_{i},z_{i})$ is the direction vector of $l_{i}$, pointing from the projection center $O_{C}^{l}$ to $p_{i}$; and $P_{i}(X_{i},Y_{i},Z_{i})$ is the desired projection point. The equation for the intersection point is as follows:

(9) $$\{\begin{array}{c} \vec{P1,P_{i}}⊥\vec{n}\\ \vec{p_{i},P_{i}}\;//\;\vec{l} \end{array}\Rightarrow \{\begin{array}{c} (X_{i}-X_{P1},Y_{i}-Y_{P1},Z_{i}-Z_{P1})\cdot (n_{x},n_{y},n_{z})=0\\ \frac{X_{i}-x_{i}}{x_{i}}=\frac{Y_{i}-y_{i}}{y_{i}}=\frac{Z_{i}-z_{i}}{z_{i}}=\lambda \end{array}$$

where λ is the scale factor. Let $F=x_{i}n_{x}+y_{i}n_{y}+z_{i}n_{z}$, F≠0; the coordinates of the projection points obtained from the above equation are as follows:

(10) $$X_{i}=\left(x_{i}(X_{P1}n_{x}+n_{y}(Y_{P1}-y_{i})+n_{z}(Z_{P1}-z_{i}))+x_{i}(y_{i}n_{y}+z_{i}n_{z})\right)/F$$

(11) $$Y_{i}=\left(y_{i}(n_{x}(X_{P1}-x_{i})+Y_{P1}n_{y}+n_{z}(Z_{P1}-z_{i}))+y_{i}(x_{i}n_{x}+z_{i}n_{z})\right)/F$$

(12) $$Z_{i}=\left(z_{i}(n_{x}(X_{P1}-x_{i})+n_{y}(Y_{P1}-y_{i})+Z_{P1}n_{z})+z_{i}(x_{i}n_{x}+y_{i}n_{y})\right)/F$$

The 3D reconstruction of crack edges and skeletons is accomplished through the utilization of Equations (10)–(12). The morphological length of the crack is determined by calculating the cumulative Euclidean distance between adjacent skeleton points, while the width at each skeleton point is obtained by computing the Euclidean distance between the pair of two edge points closest to that point. Each skeleton point corresponds to a specific crack width, from which the maximum crack width is obtained.

## 3. Training FCN

### 3.1. Crack Segmentation Database

**Figure 7:**
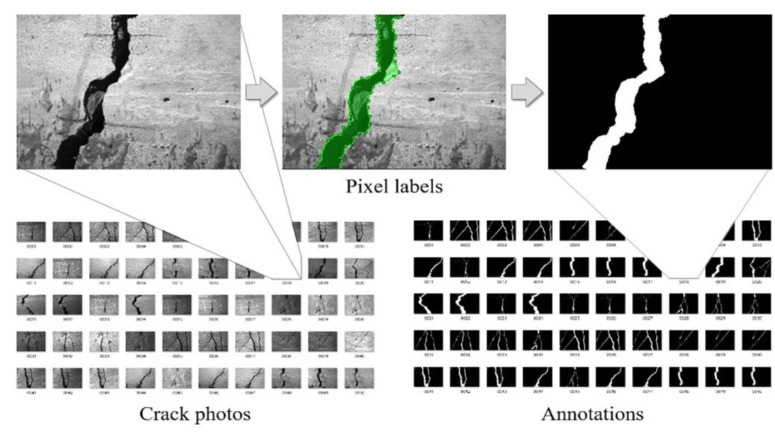
Pixel-level labeling process.

To train the FCN models, 50 photos of cracked concrete taken using a smartphone with a resolution of 4032 × 3024 × 3 and saved in JPG format are manually labeled at the pixel level using the MATLAB^R^ tool Image Labeler. Figure 7 depicts this labeling process, in which logical variables 0 and 1 are, respectively, assigned to background and crack pixels through pixel labels, with annotations saved in PNG-8 format. Subsequently, 110 images are cropped from these photos, each featuring either a crack or an intact background with 448 × 448 pixel resolution. These images, along with 334 web images of the same resolution, undergo data augmentation techniques including horizontal and vertical flips, resulting in a total of 1332 images. According to the fivefold cross-validation principle, the generated images are randomly divided into training, validation and test sets with 998, 110 and 224 images, respectively, in each set. Notably, a network trained on small-sized images can scan any image larger than that designed size [36]. Therefore, the randomly selected images and their annotations are resized to 224 × 224 pixels prior to being fed into the models.

### 3.2. Implementation Parameters

The learning rate plays a pivotal role in balancing convergence speed and stability in training a CNN. In order to choose an appropriate initial value for this key hyperparameter, three sets of models are meticulously trained, each with distinct initial learning rates: 0.001, 0.0001 and 0.00001, respectively. Throughout these training sessions, exponential stepwise decay, a common technique for annealing learning rates, is employed post epochs to reduce oscillations in the loss function around the global optimum. The decay function is as follows:

(13) $$\eta_{t}=\eta_{0}\times r_{d}{}^{\left\lfloor \frac{t}{t_{\max}}\right\rfloor }$$

where the initial learning rate is denoted by $\eta_{0}$, $r_{d}$ is the decay rate with t as the current count of iterations and $t_{\max}$ as the preset iterations for decay. ⌊⋅⌋ represents the floor operation, returning the largest integer not greater than the input value.

To assess the discrepancy between the prediction and the ground truth, cross entropy is utilized as the loss function on pixels. With exponential decay rates set to $\beta_{1}=0.9$ and $\beta_{2}=0.999$, the Adam optimizer is then run for training loss optimization by iteratively updating the model parameters. The FCN models are trained with 20 epochs, and the batch size is set to 2 (taking into account the limitations of GPU memory). In addition, a dropout rate of 0.5 is implemented to activate only half of the hidden nodes or feature detectors during each iteration, thereby weakening their interactions and effectively preventing overfitting [83,84].

### 3.3. Model Initialization and Evaluation Metrics

To expedite and optimize the learning efficiency, a model-based transfer learning strategy [85] is adopted instead of training from scratch. Following this strategy, the weights and biases of the encoder network are initialized by pre-trained VGG19. Moreover, the weights of all the deconvolutional layers in the decoding module are initialized by the truncated normal distribution with a mean of 0 and standard deviation of 0.01, and their biases are initialized as constant zero vectors.

It is widely acknowledged that pixel-level crack detection is essential to classify pixels of the input image as either a crack (positive) or the background (negative). Therefore, four cases may occur, which are outlined below:

- True Positive (crack pixels classified as crack pixels);
- False Negative (crack pixels classified as background pixels);
- False Positive (background pixels classified as crack pixels);
- True Negative (background pixels classified as background pixels).

To comprehensively evaluate the crack segmentation, three key statistical metrics are introduced: precision, recall and F1 score. These metrics are defined as follows:

(14) $$\mathrm{Precision}=\frac{\mathrm{TP}}{\mathrm{TP}+\mathrm{FP}}$$

(15) $$\mathrm{Recall}=\frac{\mathrm{TP}}{\mathrm{TP}+\mathrm{FN}}$$

(16) $$F1-\mathrm{score}=\frac{2\times \mathrm{Precision}\times \mathrm{Recall}}{\mathrm{Precision}+\mathrm{Recall}}$$

where TP, FP and FN denote the number of pixels with True Positives, False Positives and False Negatives in the predicted outcomes, respectively.

### 3.4. Training Results and Discussion

The proposed encoder–decoder FCN is implemented on Windows 10 using Python 3.5 for programming and TensorFlow 1.4 and NumPy 1.16 for building the virtual environment. All numerical experiments are performed on a desktop computer (GPU: NVIDIA GeForce GTX 1060 GPU Ti, RAM: 8 GB, CPU: Intel^®^ Core^TM^ i5-8400 CPU@2.8 GHz). With the aforementioned training method and experimental configuration, the recorded training time for each model is approximately 9 h on average after 9980 iterations, and it takes about 250 ms for a trained model to process a 448 × 448-pixel image.

**Figure 8:**
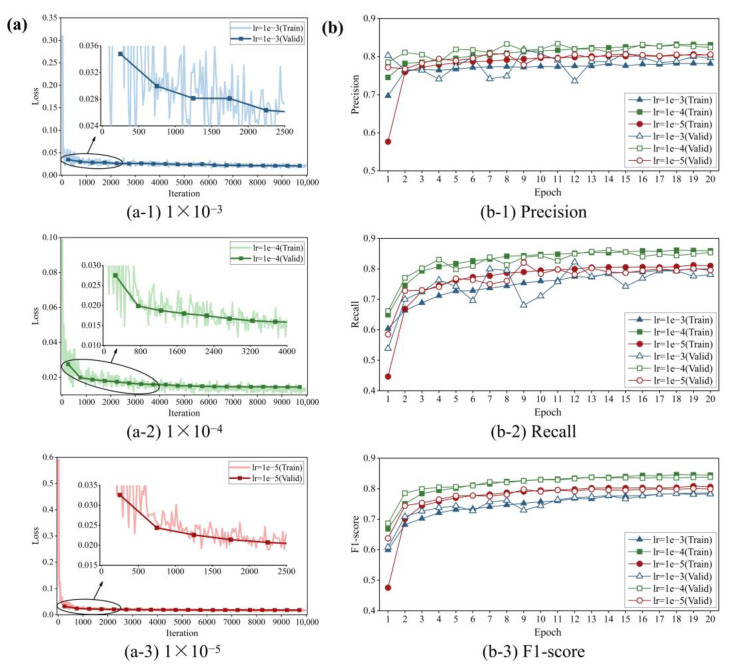
(**a**) Training and validation losses over iterations: (**a-1**) 1 × 10^−3^, (**a-2**) 1 × 10^−4^ and (**a-3**) 1 × 10^−5^. (**b**) Three evaluation metrics under epochs: (**b-1**) precision, (**b-2**) recall and (**b-3**) F1 score.

The training and validation losses at each learning rate are illustrated in Figure 8a. It can be intuitively seen that the loss value corresponding to Figure 8(a-2) exhibits the fastest convergence and ultimately stabilizes within 0.014, resulting in best training effect. The loss curves associated with the other two learning rates, i.e., 1 × 10^−3^ and 1 × 10^−5^, also demonstrate satisfactory convergence results, remaining stable at around 0.021 and 0.018, respectively, which are sufficient for attaining global optimization.

The average precision, recall and F1 score under epochs during training and validation processes at different learning rates are displayed in Figure 8b. These indicator curves climb rapidly in the first two epochs (nearly 1000 iterations), which, along with the observed plummet in training loss, demonstrates the efficacy of the transfer learning. Then, the convergence occurs after 16 epochs. Throughout the training process, the green curves with the square symbols consistently remain at the uppermost part of Figure 8(b-1)–(b-3), intuitively reflecting the exceptional performance of the FCN with an initial learning rate of 1 × 10^−4^. The highest values (not all from the same epoch) are further selected from the training and validation averages, and these results are summarized in Table 2. As can be seen from the table, 1 × 10^−4^ is the optimal learning rate, and its corresponding FCN model not surprisingly achieves the highest precision, recall and F1 score at 83.85%, 85.74% and 84.14%, respectively, highlighted in bold. Therefore, this model is used for crack segmentation.

To test the effectiveness of the proposed FCN in detecting cracks of various morphological types and background complexities, the crack images in the test set are pre-divided into four categories. (Ⅰ) Hairline crack: the cracks are narrowly developed and susceptible to changes in illumination, often resulting in fuzzy or discontinuous patterns. (Ⅱ) Block crack: the crack region exhibits a blocky pattern and occupies a significantly substantial portion of the image. (Ⅲ) Intersecting crack: the interconnected cracks show an intricate morphology. (Ⅳ) Complex background crack: the cracks in backgrounds with complex textures, speckling, shadows caused by uneven lighting, or clutter are challenging to discern through traditional methods.

**Figure 9:**
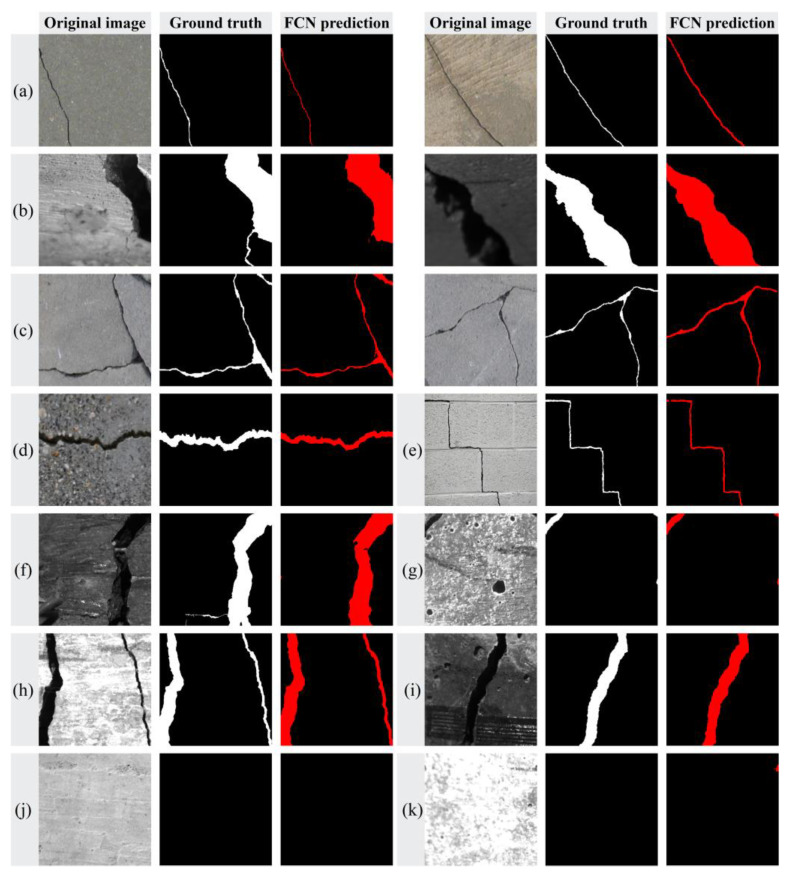
FCN predictions: (**a**) hairline crack; (**b**) block crack; (**c**) intersecting crack; (**d**) complex background crack (mottling); (**e**) complex background crack (interference); (**f**) complex background crack (clutter); (**g**) complex background crack (void); (**h**) different light condition (overexposure); (**i**) different light condition (uneven illumination); (**j**) intact surface (correct sample); and (**k**) intact surface (some pixels are False Positives).

Figure 9 depicts the FCN predictions of the above four crack types. Figure 9a–c demonstrates the segmentation results for different types of crack morphologies. The test results indicate that the proposed model exhibits good performance in accurately segmenting hairline cracks, block cracks and intersecting cracks. The segmentation of cracks under diverse and challenging conditions, including complex backgrounds and varied lighting scenarios, is also tested and compared (Figure 9e–i). In addition, Figure 9j,k display the prediction results for intact surfaces. The results demonstrate the robustness of the proposed model in handling various noise interference. Therein, the predictions of Figure 9a,c,d,g–j exhibit a significant level of agreement with ground truth. However, there are minor inaccuracies in Figure 9b (the left sample) and Figure 9f, which might be attributed to the insufficient variation in gradient of pixel values, leading to oversight of the microcracks located at the bottom. In Figure 9k, a few pixels of the backgrounds are falsely classified as cracks, possibly due to the combined interference of overexposure and overlapping black markings.

**Figure 10:**
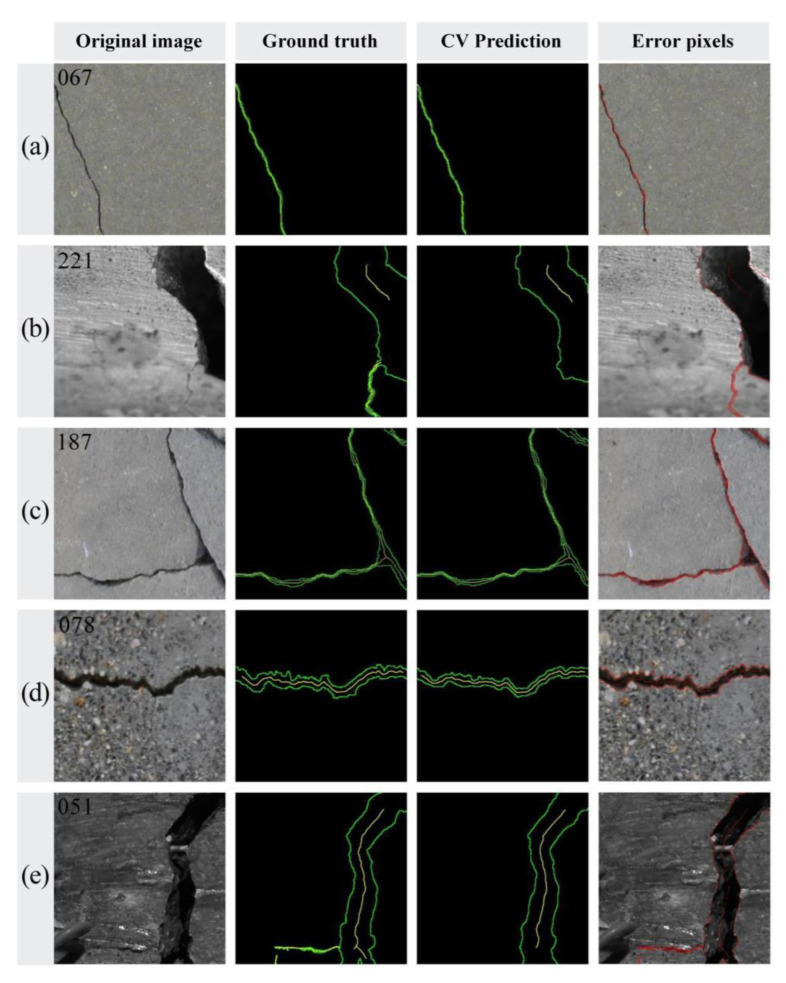
Extracted crack morphologies (The green lines represent the detected crack edges and the yellow lines represent the detected crack skeletons.): (**a**) hairline crack; (**b**) block crack; (**c**) intersecting crack; (**d**) complex background crack (mottling); and (**e**) complex background crack (clutter).

Although the overall accuracy of FCN segmentation is somewhat compromised due to these omissions in detail, the extracted crack edges and skeletons still maintain an acceptable level of validity (Figure 10).

## 4. Experiment

In this section, an experiment is conducted to detect cracks in concrete specimens subjected to static load tests, with the aim of verifying the practical feasibility of the proposed method. The damaged concrete beams and slabs are neatly arranged on one side of the laboratory, and the binocular photography system is positioned approximately 0.2 m away from these cracked concretes. The aperture is adjusted accordingly to optimize exposure and capture cracks in natural indoor lighting, while simultaneously recording the manually measured values of both crack width gauges with a 0.01 mm accuracy and crack ruler as reference values for the actual crack width.

**Figure 11:**
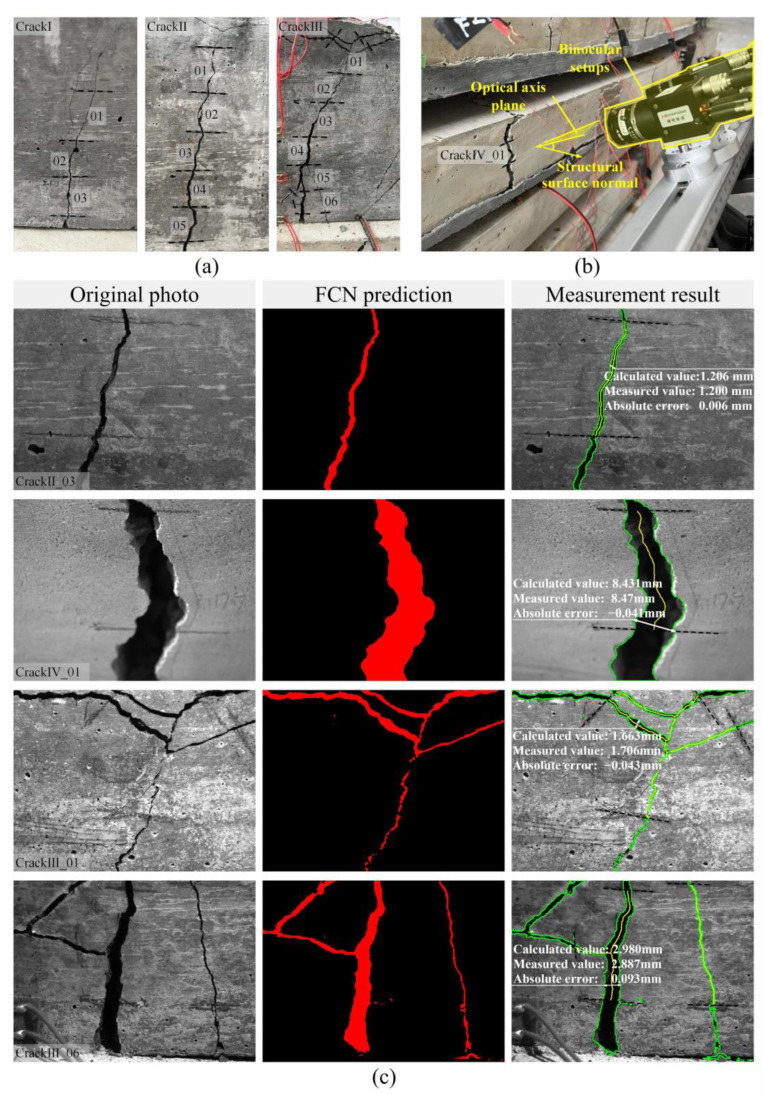
Concrete crack detection and measurement experiment: (**a**) divided crack fragments (the crack segment numbering corresponds to the numbering in the bottom left corner of the crack images in (**c**)); (**b**) binocular device overlooking a crack; and (**c**) visualization of the results for certain fragments.

The experimental setup is illustrated in Figure 11, and a total of four cracks have been identified. Among them, three complex background cracks, designated as CrackI, CrackII and CrackIII, respectively, originating from the same beam specimen are artificially divided into multiple fragments before photographing, that is, the crack areas between black dashed lines in Figure 11a, to enhance the quantity of control groups for comparison. Additionally, as shown in Figure 11b, the fourth block crack is denoted as CrackIV_01, which is observed on a slab specimen and shot from the overhead perspective at a certain angle between the optical axis plane and the structural surface normal. The measured results are summarized in Table 3, Table 4 and Table 5, where the maximum error is 0.144 mm, corresponding to a relative error of 36.0%. This is attributed to the non-negligible prediction bias of FCN for CrackⅠ_01. Hence, it is imperative to further optimize the performance of FCN for detecting hairline cracks.

Figure 11c presents the visible outcomes of certain crack fragments, among which the refined red region effectively demonstrates the generalization capability of our FCN, while the low error level further substantiates the validity of the proposed measurement method. Specifically, CrackⅡ_03 has achieved the most accurate quantification, with an error of only 0.006 mm. As anticipated, CrackⅣ_01, exhibiting a calculated error of −0.069 mm, confirms the binocular vision-based approach’s capability to maintain high measurement accuracy even under oblique shooting conditions, thereby highlighting its superiority over the monocular vision method in terms of shooting posture. Although the morphology of CrackⅢ_06 is successfully extracted despite the interference of the strain gauge wire and the shadow caused by this wire in the lower left corner, the associated error exhibits a substantial increase in comparison to CrackⅢ_01, reaching 0.093 mm. One possible explanation for this is that the uneven concrete surface renders the proposed method inapplicable. Apart from displaying maximum values of crack width, their specific location are also indicated through white bidirectional arrows, thereby offering a valuable reference for re-inspection.

## 5. Conclusions and Discussion

In this paper, a non-contact method for detecting and measuring cracks is proposed by combining a semantic segmentation network, specifically the encoder–decoder FCN, with binocular stereo vision, which achieves a balance between efficiency and accuracy. According to the research results, the following conclusions can be drawn:

1. To fit the ground truth to the fullest extent, the proposed FCN adopts the encoder–decoder structure and skip connections to enable enhanced focus on details during crack segmentation. The optimal FCN model is fine-tuned using a training dataset consisting of 1108 concrete surface images with a resolution of 448 × 448 pixels, resulting in satisfactory levels for all three evaluation metrics: precision at 83.85%, recall at 85.74% and F1 score at 84.14%. These results demonstrate that the proposed FCN can accurately detect cracks at the pixel level. Since a plate is a commonly used substructure in civil engineering, an experiment of a steel plate is carried out to validate the feasibility of the proposed methodology.
2. An integrated CV procedure is specifically designed to extract the edges and skeletons of cracks from binary graphs predicted by FCN, with the aim of preparing data for crack measurements. The performance of the CV procedure is subsequently assessed on FCN predictions of various types of cracks in the test set, demonstrating that its output is both acceptable and effective. Moreover, skeletonization results exhibit a higher level of adherence to the actual crack topology in regions that are distant from the image boundary.
3. The proposed method is applied to quantitatively evaluate the cracking of concrete specimens in real-life scenarios, with a comparison made against manual inspection results. The experimental results demonstrate that our FCN possesses remarkable generalization capability, and the binocular measurement method can also control errors at a low level, thereby ensuring both robustness in detection and accuracy in measurement. For crack width, the maximum error is 0.144 mm, while the mean relative error stands at 5.03%, thus confirming the feasibility of the proposed method.
4. The experiment also involves an overhead shot of a target crack through the binocular photography system. The calculated error of −0.041 mm, along with its corresponding relative error of −0.8%, validates the high level of accuracy achieved by the binocular vision-based measurement method even under tilted shooting conditions, emphasizing its superiority over the monocular vision method and making it more suitable for implementation on remotely operated piggyback platforms, such as UAVs or robots.

However, there are still some limitations to this research. Future studies should aim to integrate advanced algorithms like attention mechanisms and EfficientNet to further enhance the model’s performance. Additionally, the incorporation of advanced feature matching algorithms like LightGlue promises to yield more precise three-dimensional reconstructions of cracks. In practical terms, the proposed binocular photography system requires an external power source of 5V or higher. It is necessary to optimize the energy management strategy for the entire detection system. This may involve reducing standby power consumption and employing dynamic programming to determine the optimal flight path of UAVs. This research, currently focused on crack segmentation and measurement, should expand to include other surface defects like delamination and spalling in future studies, broadening its scope and real-world applicability.

## Figures and Tables

**Table 1:** Detailed specifications of the binocular system.

Component	Model	Specification
CCD grayscale camera@2	MV-EM120M	Sensor resolution: 1280 × 960 pixels Pixel size: 3.75 × 3.75 (μm) Size: 29 × 35 × 48.9 (mm) Weight: 50 g
Industrial fixed-focus lens@2	BT-118C1620MP5	Focal length: 16 mm Size: φ27.2 × 26.4 (mm) Weight: 75 g

**Table 2:** Model performance at different learning rates.

Initial Learning Rate (×10^−4^)	Highest Precision (%)	Highest Recall (%)	Highest F1 Score (%)
0.1	80.48	80.67	80.47
1	**83.10**	**85.74**	**84.14**
10	79.53	79.84	78.43

Note: The values highlighted in bold represent the best training results of our FCN.

**Table 3:** Results of maximum width measurement for CrackⅠ, CrackⅢ_06 and CrackⅣ_01.

Measurement Result	CrackⅠ_01	CrackⅠ_02	CrackⅠ_03	CrackⅢ_06	CrackⅣ_01
Calculated value (mm)	0.544	0.981	1.993	2.980	8.431
Reference value (mm)	0.400	1.045	2.106	2.887	8.5 *
Error (mm)	0.144	−0.064	−0.113	0.093	−0.069
Relative error	36.0%	−6.1%	−5.4%	3.2%	−0.8%

Note: * indicates that the reference value is obtained by the crack ruler.

**Table 4:** Results of maximum width measurement for CrackII (01–05).

Measurement Result	CrackⅡ_01	CrackⅡ_02	CrackⅡ_03	CrackⅡ_04	CrackⅡ_05
Calculated value (mm)	0.803	1.601	1.206	1.722	2.168
Reference value (mm)	0.836	1.613	1.200	1.743	2.153
Error (mm)	−0.033	−0.012	0.006	−0.021	0.015
Relative error	−3.9%	−0.7%	0.5%	1.2%	0.7%

**Table 5:** Results of maximum width measurement for CrackIII (01–05).

Measurement Result	CrackⅢ_01	CrackⅢ_02	CrackⅢ_03	CrackⅢ_04	CrackⅢ_05
Calculated value (mm)	1.663	1.124	2.081	2.067	2.165
Reference value (mm)	1.706	1.045	2.090	2.026	2.129
Error (mm)	−0.043	0.079	−0.009	0.041	0.036
Relative error	−2.5%	7.6%	0.4%	2.0%	1.7%

## Data Availability

Data are contained within the article.
